# Opening of Wesley Memorial Hospital

**Published:** 1905-09

**Authors:** 


### OPENING OF WESLEY MEMORIAL HOSPITAL.

The Wesley Memorial Hospital was opened Wednesday, August 16th, there being present more than one thousand interested in the hospital, who were well repaid for their coming by the able addresses of Dr. J. C. Todd and Bishop W. A. Candler.

The hospital is located on the corner of Courtland and Houston streets, and is a large building with three stories and a basement.

The equipment throughout is complete, with forty beds conveniently arranged and scrupulously clean. The automatic elevator, as far as we know, is unique in this part of the country. It carries one to the floor desired when the door is closed and the proper button pressed, and stops of its own accord.

The operating-room is large, well lighted, and is fitted up with the most modern plumbing, a first-class sterilizer, and every appointment necessary for conducting an aseptic operation.

Miss Stella Shipman is superintendent of a corps of nurses, to be increased as needed.

Dr. Stevens is the resident physician.

Much credit is due Bishop Candler, whose untiring efforts have made Wesley Memorial a modern hospital.

---

DR. E. G. BALLINGER has been made associate editor and assistant business manager of this journal. Dr. Ballinger is a graduate of the University of Maryland, served as house physician of the University Hospital and perfected himself in his specialty, genitourinary diseases, through post-graduate work in Johns Hopkins. He holds examining board certificates from the States of Maryland, North Carolina, South Carolina and Georgia and is a young man of fine intellect and unusual energy.

